# Changes of soil carbon along precipitation gradients in three typical vegetation types in the Alxa desert region, China

**DOI:** 10.1186/s13021-024-00264-2

**Published:** 2024-06-17

**Authors:** Xinglin Zhu, Jianhua Si, Bing Jia, Xiaohui He, Dongmeng Zhou, Chunlin Wang, Jie Qin, Zijin Liu, Li Zhang

**Affiliations:** 1 Key Laboratory of Eco-Hydrology of Inland River Basin, Northwest Institute of Eco-Environment and Resources, Chinese Academy of Sciences, Lanzhou, China; 2 University of Chinese Academy of Sciences, Beijing, China; 3 Faculty of Resources and Environment, Baotou Teachers’ College, Inner Mongolia University of Science and Technology, Baotou, China; 4 Alxa Left Banner Public Service Center, Alxa League, 750306 China

**Keywords:** Soil inorganic carbon, Soil organic carbon, Precipitation gradient, Arid desert area, Alxa Plateau

## Abstract

The changes and influencing factors of soil inorganic carbon (SIC) and organic carbon (SOC) on precipitation gradients are crucial for predicting and evaluating carbon storage changes at the regional scale. However, people’s understanding of the distribution characteristics of SOC and SIC reserves on regional precipitation gradients is insufficient, and the main environmental variables that affect SOC and SIC changes are also not well understood. Therefore, this study focuses on the Alxa region and selects five regions covered by three typical desert vegetation types, *Zygophyllum xanthoxylon* (ZX), *Nitraria tangutorum* (NT), and *Reaumuria songarica* (RS), along the climate transect where precipitation gradually increases. The study analyzes and discusses the variation characteristics of SOC and SIC under different vegetation and precipitation conditions. The results indicate that both SOC and SIC increase with the increase of precipitation, and the increase in SOC is greater with the increase of precipitation. The average SOC content in the 0–300cm profile is NT (4.13 g kg^−1^) > RS (3.61 g kg^−1^) > ZX (3.57 g kg^−1^); The average value of SIC content is: RS (5.78 g kg^−1^) > NT (5.11 g kg^−1^) > ZX (5.02 g kg^−1^). Overall, the multi-annual average precipitation (MAP) in the Alxa region is the most important environmental factor affecting SIC and SOC.

## Introduction

Soils are the largest carbon pool in terrestrial ecosystems, with soil carbon stocks accounting for more than quadruple the biological carbon stock and more than triple the atmospheric carbon stock, respectively [39, 93]. Soil carbon stocks depend mainly on changes in soil inorganic carbon (SIC) and organic carbon (SOC) inputs and outputs, a process that is influenced by land use type, vegetation type, soil properties and microbial activity, and driven in particular by precipitation and temperature among the climatic variables. In addition, slight variations in soil carbon pools can further affect atmospheric carbon dioxide concentrations and global climate conditions [16, 33, 70, 94]. Understanding the patterns, magnitude and driving mechanisms of carbon cycling in terrestrial ecosystems remains a major challenge for Earth system science research [28, 46]. Thus, the study of changes in SIC density and SOC density over precipitation gradients and the factors influencing them is essential for predicting and assessing variations in carbon stocks at the regional scale.

Soil carbon pools can be categorized into SIC and SOC, and slight changes in both can have important impacts on regional-scale carbon balance and terrestrial ecosystem carbon cycle [4, 42, 76, 101]. SOC, which is derived from the decomposition and transformation of plant apomictic material, is more active compared to SIC, and exhibits a rapid response to environmental and climatic changes. SIC is stored mainly as dolomite (MgCO_3_) and calcium carbonate (CaCO_3_) in arid desert areas with scarce precipitation and is a more stable part of the soil carbon stock [25, 54, 55, 63]. SIC and SOC content also differed significantly in the driving mechanisms at different soil depths [34, 49, 77, 80]. Major climatic variables such as Multi-year average precipitation (MAP) and Multi-year average temperature (MAT) control Soil moisture content (SMC) dynamics while affecting the distribution and growth of vegetation, thus contributing to the dominant role of vegetation in the variation of SIC and SOC [45, 60]. Vegetation type, root dispersion, and soil water content all had an impact on SOC decomposition and buildup at various levels of the soil, while soil type, soil water content, and vegetation type also had an impact on SIC dissolution and leaching. However, particularly in dry desert ecosystems, it is unknown how much of role factor like precipitation, temperature, soil type, soil characteristics, etc. have in influencing soil carbon accumulation. Precipitation plays a decisive role in determining net primary productivity and ecosystem structure in arid ecosystems, which in turn may influence the accumulation of SIC and SOC through abiotic or biotic factors associated with soil particulate matter deposition, vegetation productivity, and organic matter decomposition [10, 14, 92, 98]. Most studies indicate that SOC typically rises with precipitation, but that SOC tends to exhibit a propensity to fall with soil depth because of biological factors such as uneven vegetation production and inputs of litter [43, 52, 72, 82]. Due to the scant amount of data, it is unclear how the SIC changes with soil depth and along the gradient of precipitation. As precipitation increased, SIC was reported to exhibit both increasing [81] and decreasing [61] results. Additionally, researchers have noted changes in the vertical distribution of SIC that are generally stable [24], decreasing [81] and increasing [73–75, 79]. The spatial distribution of SIC and SOC profiles and their interaction with precipitation gradient are yet understudied aspects of the dry desert region, where precipitation is infrequent and vegetation is scant.

Arid deserts are distinguished by a lack of flora and a SOC content that is relatively low. The SIC in arid deserts has long been disregarded in the study of the terrestrial ecosystem carbon cycle because some academics once thought that it was essentially a “dead reservoir” and that its contribution to the modern carbon cycle was minuscule [65]. As a result, studies on the global carbon cycle have long ignored SIC in arid deserts. However, as the dominant form of soil carbon pool in semi-arid and arid zones, SIC may largely contribute to soil carbon loss due to climate warming [70, 94]. According to recent studies, 27% of the total CO_2_ emissions from calcareous soils are attributed to SIC-derived CO_2_ [70]. This implies that SIC is contributing more and more to the global carbon cycle. Although the SIC pool is slightly lower than the SOC pool at depths of 0–100 cm, estimates by various methods show that it is very large: 695–748 Pg C [5], 940 Pg C [17], and 950 Pg C [66], exceeding the atmospheric carbon pool (760–880 Pg C) and terrestrial biotic carbon pool (540–610 Pg C) [59]. More surprisingly, in soils at 0–200 cm depth, SIC stocks exceed 2300 Pg, which is comparable to SOC stocks (2400 Pg) [5, 93]. This makes soil inorganic carbon important in the global carbon cycle. Additionally, because the worldwide desert region accounts for nearly one-fourth of the planet's land area, its crucial significance in reducing climate change cannot be understated [77, 80]. With a total size of roughly 270,000 km^2^, Alxa is a typical dry desert region in the westernmost region of Inner Mongolia, China. With obvious zonal soil variations, the region is a transition zone from a steppe desert to a normal desert to a severely arid desert. It is currently unknown how much SIC and SOC are available in the soils of different vegetation types in this region, which has extremely spatially heterogeneous ecosystems and intricate and diverse vegetation forms. More investigation is needed to compare the impact of various vegetation types, soil types, soil features, and other factors on the distribution and density of SIC and SOC on a broad regional scale. The dynamics of SIC and SOC along the region’s climatic gradients haven’t been the subject of numerous studies. These information gaps served as the primary driving force behind our endeavor.

On this basis, this study selected three typical vegetation types (*Zygophyllum xanthoxylon* (ZX), *Nitraria tangutorum* (NT), and *Reaumuria songarica* (RS)) from arid areas on five plots of natural precipitation gradient in the Alxa Plateau. By comparing the distribution characteristics and trends of SIC and SOC of three vegetation types at depths of 0–300 cm, we aim to understand the changing trends of SOCD and SICD with precipitation gradients in the Alxa region, as well as the main driving factors of SOCD and SICD changes at different soil depths. Provide the best solution for ecological afforestation and soil carbon sequestration under different precipitation gradients in arid desert areas based on the differences in soil carbon density among vegetation types.

## Materials and methods

### Study area and transect description

With a total area of 270,000 square kilometers, the Alxa Plateau is situated in the southwest of the Mongolian Plateau in the heart of interior Asia. The Afro-Asian Desert Zone, a mesothermal to warm-temperate transition to a desert characterized by shrubs and semi-shrubs, includes the Alxa Plateau, which is situated on its eastern flank. The geographic coordinates are between 97°10′–106°53′ East and 37°24′–42°47′ North. The landscape of the Alxa Plateau is dominated by high plains, while mountains, hills, and deserts are widely distributed. The Ulan Buh, Tengger, and Badain Jilin Deserts are three of China’s eight principal deserts, making up around 30% of the Alxa Plateau's overall area. The surface of the three major deserts of Badain Jilin, Tengger, and Ulan Buhai is covered by a deep and loose sand layer. Alxa League is an interior plateau with a continental monsoon climate that is far from the sea. The geography, geomorphology, and bioclimatic conditions of the Alxa Plateau soil result in clear zonal distribution features, with brown calcic soil from the slow transition to gray desert soil and gray-brown desert soil occurring from the northwest to the southeast. There are 29.8 million acres of native shrubs in Alxa Plateau.

**Fig. 1:**
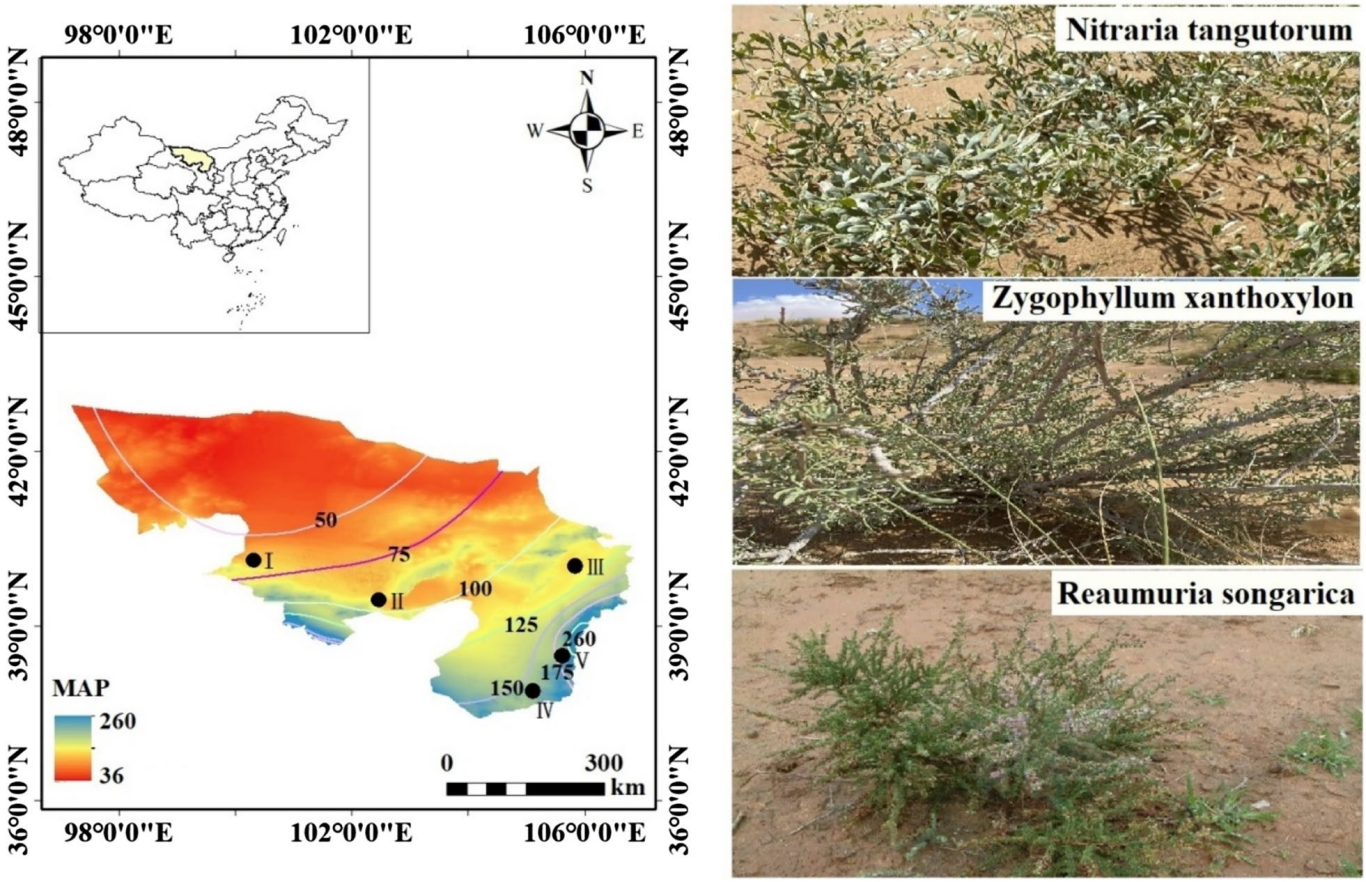
Sampling point locations, rainfall lines, and plant type diagrams, where I, II, III, IV, and V represent Alaten Chaog, Zhongquanzi, Kirantai, Toudao Lake, and Bayanhot, respectively. Precipitation data from Alxa League Meteorological Monitoring Station

In this study, the deserts and Gobi in the Alxa region were not included in the survey scope due to sparse vegetation. The five survey points selected in other areas of the Alxa region were Aratenchage (with a regional area of 19,000 square kilometers), Zhongquanzi (with a regional area of 11,717.6 square kilometers), Jilantai (with a regional area of 12,386 square kilometers), Toudao Lake (with an area of 4206 square kilometers), and Bayanhot (with an area of 5478 square kilometers). According to meteorological data in the Alxa region, there are complete records of annual precipitation and annual average temperature in these areas, and precipitation has obvious regional characteristics, as shown in Fig. 1. Meanwhile, in these areas, the distribution of overlords, red sand, and white thorns is widespread, and the vegetation between the plots is comparable. In each of the five locations, three typical types of desert vegetation were selected: bully, white thorn, and red thorn. The MAT range of these locations is 7.9 ℃to 9.1 ℃, and the MAP has increased from 36 to 260 mm. Precipitation is mainly distributed from June to September. In this study, MAP was calculated based on monitoring values from 1987 to 2015.

The soil types in this study were based on the 1:1 million “Soil Map of the People’s Republic of China” compiled and published by the National Soil Census Office in 1995 (https://www.Resdc.cn). Based on the vectorization of the Alxa map, obtain the soil types of the five study areas. The soil types at these five sites were mobile windswept sandy soils (MWSS), grayish-brown desert soils (GBDS), grayish-desert soils (GDS), light grayish-calcareous soils (LGCS), and brownish-calcareous soils (BCS). Each site's sampling points for the various vegetation kinds were close to one another, and the soil's texture and type were generally constant. Basic facts about the five sites are listed in Table 1.

**Table 1:** The basic description of the studied sites

Site	Alaten Chaog		Zhongquanzi		Kirantai		Toudao Lake		Bayanhot	
Soil type	MWSS		GBDS		GDS		LGCS		BCS	
MAP(mm)	65.6		84.6		120.8		149		206.7	
MAT(℃)	8.7		9.1		8.9		8.2		7.9	
Altitude (m)	1462		1257		1328		1480		1309	
Plant type	MH(cm)	CR(%)	MH(cm)	CR(%)	MH(cm)	CR(%)	MH(cm)	CR(%)	MH(cm)	CR(%)
OD	53.2	5.3	56.7	6.1	73.3	8.3	95.8	14.5	123.2	17.3
NT	13.6	4.3	15.2	5.2	17.3	9.3	16.8	13.5	17.2	19.3
RS	30.4	5.5	35.2	5.8	32.7	8.3	38.8	12.5	53.2	18.3

WH, CR distributions indicate the mean plant height and mean plant cover at the three sampling sites

### Soil sampling and measurements

**Fig. 2:**
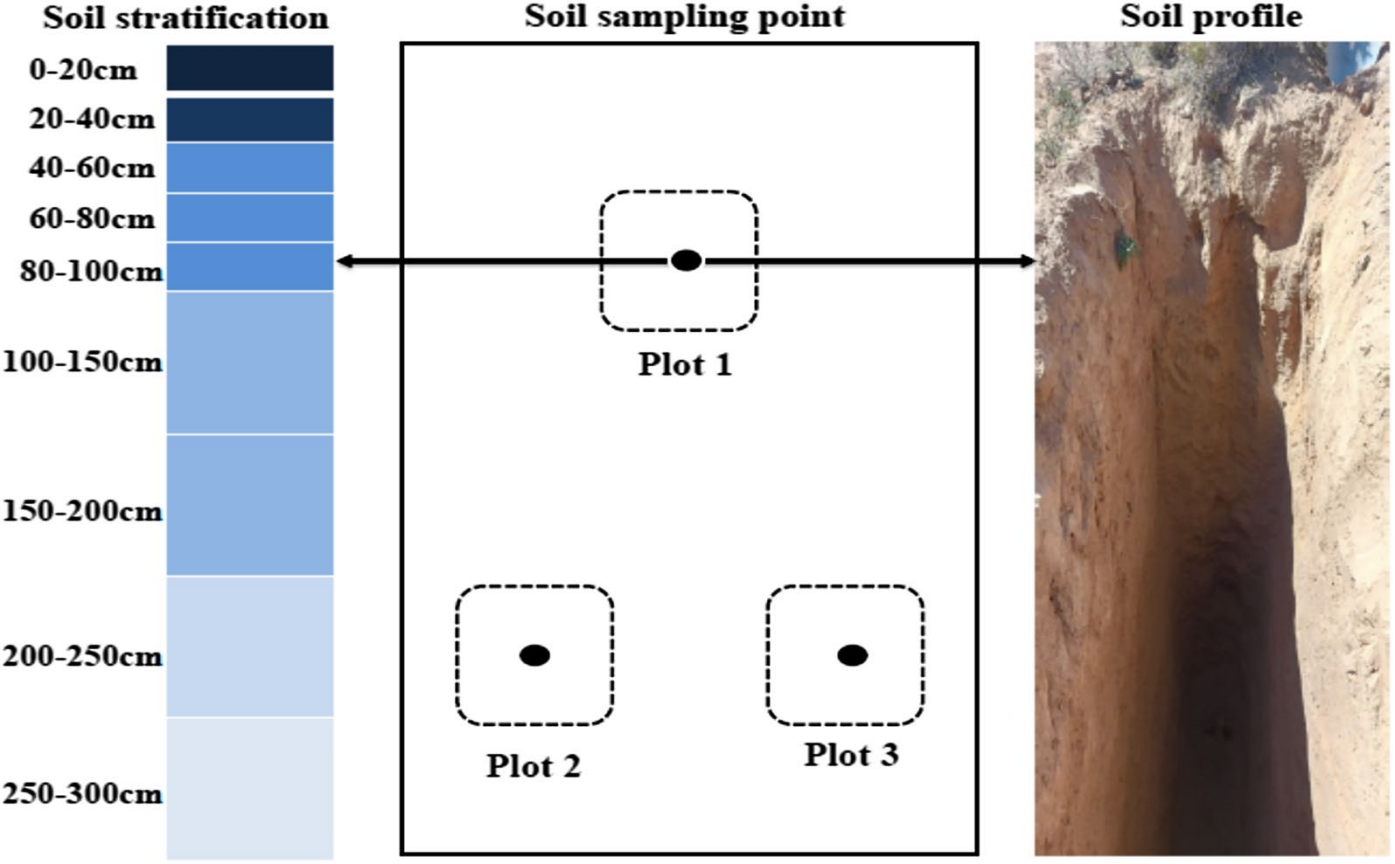
Layout of Soil Sampling Points and Soil Stratification Schematic

Before conducting soil sampling surveys, we first select areas far away from human activities to conduct experiments. Then, a vegetation survey will be conducted, and areas with three typical vegetation types that do not overlap with each other will be used as sample plots for soil sampling. Vegetation plots with good growth status and similar community density will be selected for soil sampling. This ensures that soil samples are not affected by human activities and cross vegetation. In order to replicate the soil samples, three 5 × 5 m plots were set in the middle of each vegetation type. The way each vegetation type is surveyed is based on the classical rules for vegetation surveys. There were nine layers of soil samples in each plot: 0–20 cm, 20–40 cm, 40–60 cm, 60–80 cm, 80–100 cm, 100–150 cm, 150–200 cm, 200–250 cm, and 250–300 cm. Shallow soil samples from 0 to 100 cm were not mixed, and deeper soil samples from 100 to 300 cm were mixed according to a 50 cm layer. Using stainless steel cutting rings (100 cm^3^ in volume), a soil profile was drilled to a depth of 300 cm in the middle of each stand to gather the soil bulk density (BD) of the undisturbed soil cores. The layout of soil sampling points and soil stratification are shown schematically in Fig. 2.

To eliminate stones or roots larger than 2 mm, the treated and air-dried soil samples were sieved. It was then pulverized in a ball mill and put through a soil filter with a mesh size of 100. The K_2_Cr_2_O_7_–H_2_SO_4_ oxidation method was used to measure the SOC content, and a modified pressure transducer method was used to measure the SIC content. The pH was calculated using the electrode method, and BD represents the soil's dry weight per volume after drying. For SMC, the soil’s fresh weight was measured first, and once it had dried, its dry weight was measured.

### Calculation of SOC and SIC density and data analysis

The following equation was used to determine SICD or SOCD (kg m^−2^) from soil bulk weight and inorganic or organic carbon content:

1 $$SICD = \, SIC_{i} \times BD_{i} \times D_{i} \times (1 - G_{i} {)}/100$$

2 $$\, SOCD = \, SOC_{i} \times BD_{i} \times D_{i} \times (1 - G_{i} )/100$$

where SIC_i_ (g kg^−1^) represents the soil's inorganic carbon content, SOC_i_ (g kg^−1^) represents its organic carbon content, BD_i_ (g cm^−3^) represents its bulk density, and D_i_ (cm) represents the soil's layer thickness, G_i_ is the gravel content (> 2 mm) of soil layer D_i_.

Using one-way analysis of variance (ANOVA) and multiple comparisons in SPSS (version 23.0), the variability of SOC and SIC between vegetation types or soil depths was investigated. To investigate trends in SOCD and SICD along the gradient of precipitation in Origin (2022), linear regression was utilized. Using Pearson correlation coefficients, the significance of the influencing factors as well as the link between SOC and SIC densities and environmental factors were determined. Redundancy analysis (RDA) was performed using Canoco software (version 5.0) to assess the relative contribution of environmental factors to the variability of SOC and SIC densities in different soil layers.

## Results

### Profile changes in soil carbon content of different vegetation types

**Fig. 3:**
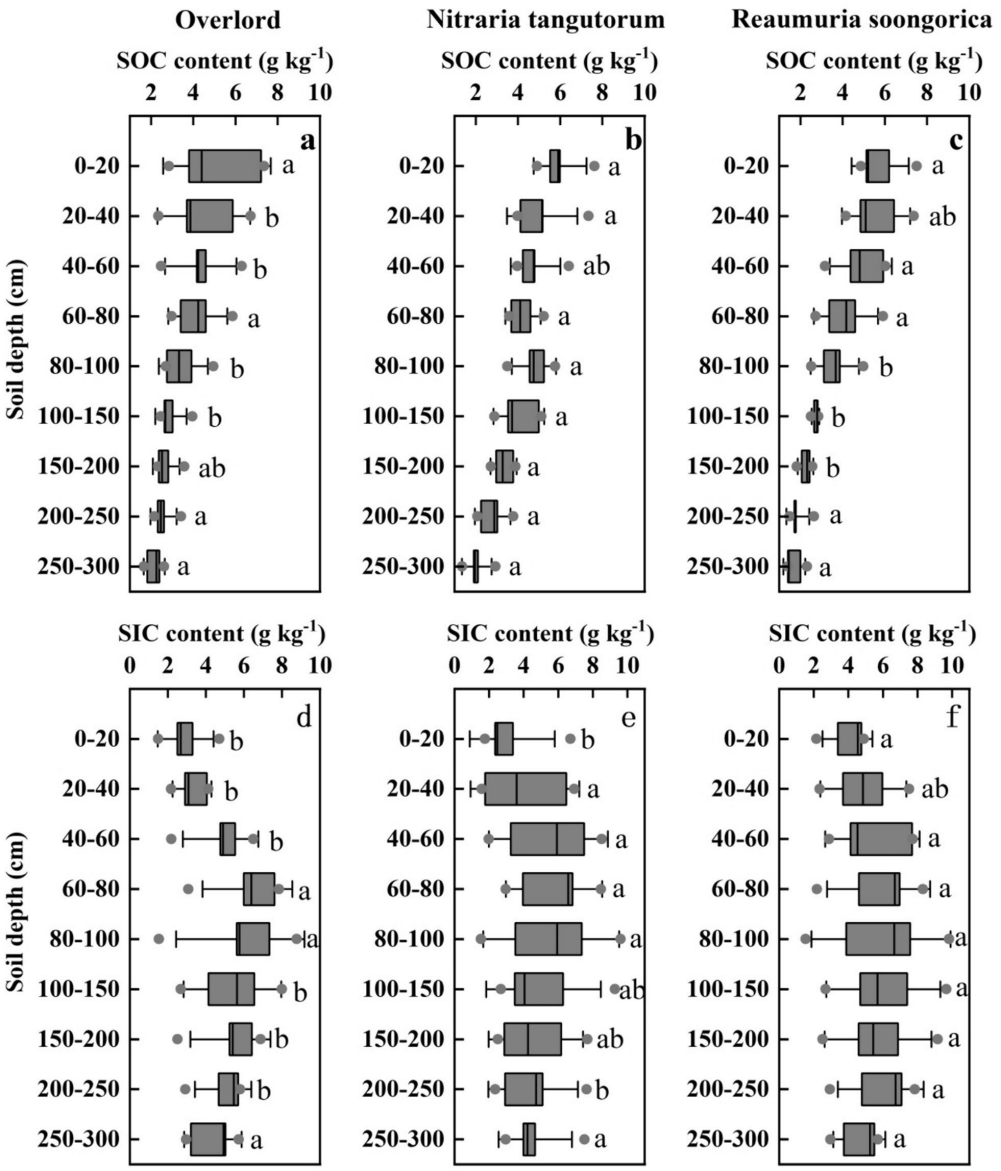
Distribution of SIC and SOC contents by soil profile for ZX, NT, and RS. Error lines show how the same plant at the same site differs in SOC and SIC. Significant changes in SIC and SOC contents of ZX, NT, and RS in the same soil layer are represented by various lower case letters (P < 0.05). **a**, **b**, **c** denote the SOC content of ZX, NT, and RS, and **d**, **e**, **f** denote the SIC content of ZX, NT, and RS, respectively

The SOC content had a substantial vertical distribution, as seen in Fig. 3 (a–c). The SOC content gradually decreased with soil depth. The SOC concentrations of OD, NT, and red sand RS revealed various significant changes in the various soil layers according to soil depth. The reason for this is that as the soil layer deepens, the ability of vegetation to increase soil carbon content gradually decreases. Mainly attributed to the differences in the distribution of litter and roots on the profile, both of which are the main sources of SOC. Firstly, the surface litter is difficult to transport to the bottom soil, making it difficult for the carbon input from litter to enter deeper soil layers. In addition, the surface root biomass of each vegetation type is higher than that of the bottom layer, and it gradually decreases with the deepening of the soil layer, resulting in a decrease in carbon input by the roots as the soil layer deepens, leading to a decrease in SOC content as the soil layer deepens. The SOC content did not differ significantly between ZX (5.12 g kg^−1^), NT (5.99 g kg^−1^), and RS (5.77 g kg^−1^) in the top soil layer (0–20 cm). The SOC content differed significantly between the profiles of ZX, NT, and RS in the soil layer of 20–200 cm. The SOC concentration in the profiles of ZX, NT, and RS did not differ noticeably within the 200–300 cm soil layer. From Table 2, the mean SOC content values for the 0–300 cm profiles were as follows: NT (4.13 g kg^−1^) is followed by RS (3.61 g kg^−1^) and ZX (3.57 g kg^−1^).

**Table 2:** Descriptive statistics of ZX, NT and RS at 0–300 cm SOC and SIC content

Soil carbon content	Plant type	Min	Max	Mean	SD	CV (%)
SOC(g kg^−1^)	ZX	2.14	5.12	3.57	1.02	0.28
	NT	2.04	5.99	4.13	1.23	0.29
	RS	1.71	5.77	3.61	1.56	0.43
SIC(g kg^−1^)	ZX	2.92	6.18	5.02	1.18	0.23
	NT	3.33	6.06	5.11	0.86	0.16
	RS	3.94	6.79	5.78	0.99	0.17

The change of SIC content in the soil profile was different from the progressive decline of SOC content, as seen in Fig. 3 (d–f).On the other hand, the SIC content displayed a pattern of minor growth followed by a steady decline. In the 0–20 cm top soil level, there was no significant difference between ZX (2.92 g kg^−1^) and NT (3.33 g kg^−1^). However, there was a significant difference with RS (3.94 g kg^−1^). Vertical distribution of SIC content of different plant types in the 20–60 cm soil profile showed that SIC content was much lower than SOC content. There was no discernible change in SIC content between ZX, NT, and RS in the 80–100 cm soil profile. From Table 2, the SIC concentration within the 100–300 cm soil profile demonstrated the same considerable variation in ZX, NT, and RS. The mean SIC content values in the 0–300 profile were in the following order: RS (5.78 g kg^−1^) > NT (5.11 g kg^−1^) > ZX (5.02 g kg^−1^).

### Differences in soil carbon density among different vegetation types

**Fig. 4:**
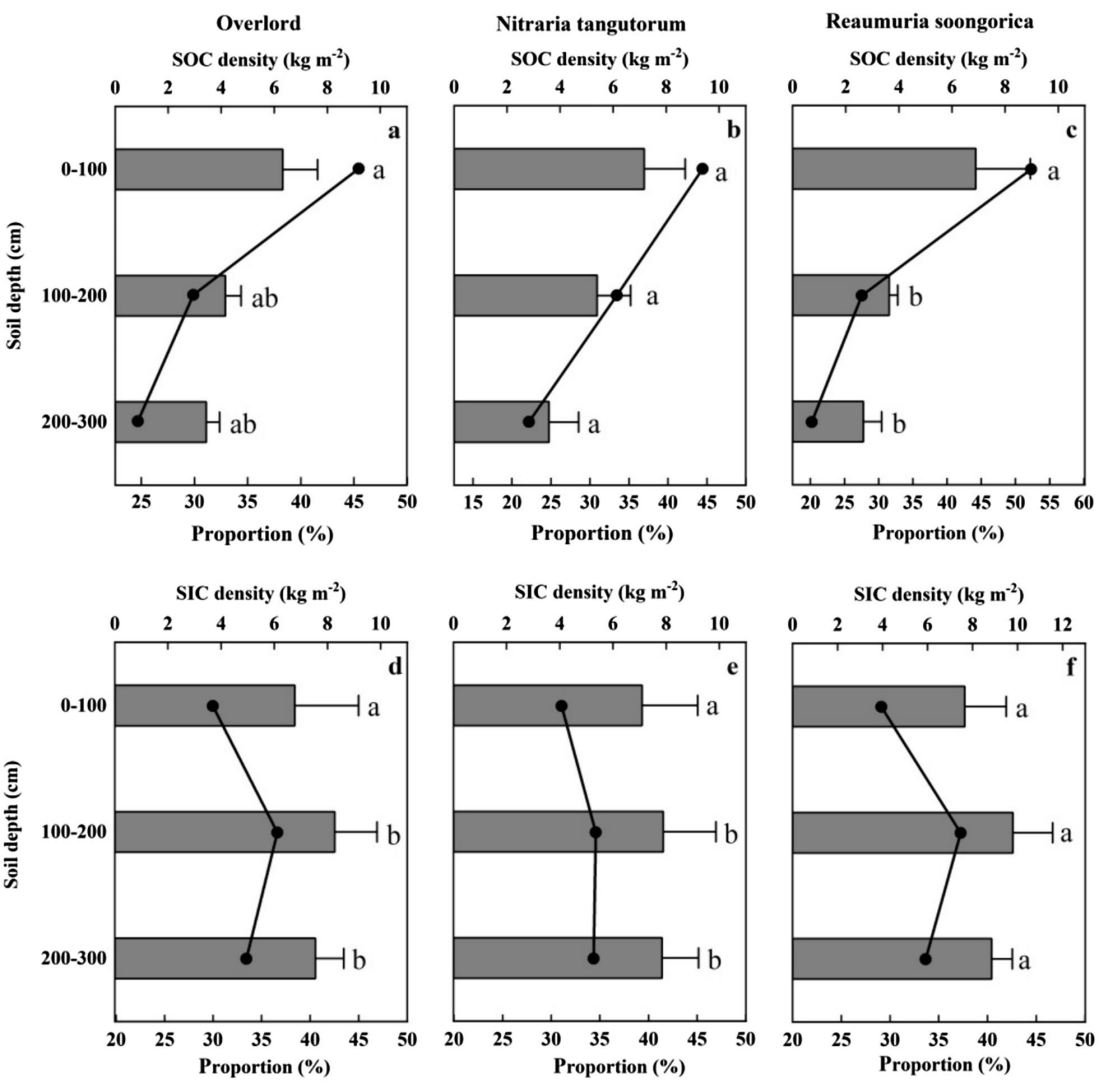
The number of SOCD and SICD of ZX, NT, and RS (bars) and their percentages (black circles) in various soil layers. For ZX, NT, and RS, different lowercase letters denote statistically significant variations in SOC and SIC densities within the same soil layer (P < 0.05). **a**, **b**, **c** denote the SOC content of ZX, NT, and RS, and **d**, **e**, **f** denote the SIC content of ZX, NT, and RS, respectively

Figure 4a–c shows the variation in ZX, NT, and RS as well as the variation in SOCD across various soil profiles. In the 0–100 cm soil layer, the SOCD differences between ZX (6.31 kg m^−2^), NT (7.17 kg m^−2^), and RS (6.89 kg m^−2^) were not statistically significant. The percentage of SOCD in the top 100 cm soil depth was ZX (45.44%), NT (44.42%), and RS (52.28%) in the 0–300 cm soil layer. At soil depths of 100–200 cm, there was a large difference in SOCD between NT (5.39 kg m^−2^) and RS (3.62 kg m^−2^), but not much difference with ZX (4.15 kg m^−2^). The percentage of SOCD at 100–200 cm soil depth in the 0–300 cm soil layer was ZX (29.88%), NT (33.43%), and RS (27.53%), respectively. At 200–300 cm soil depth, the difference in SOCD between NT (3.57 kg m^−2^) and RS (2.66 kg m^−2^) was significant, but the difference with ZX (3.43 kg m^−2^) was negligible. The percentage of SOCD at 200–300 cm soil depth in the 0–300 cm soil layer was ZX (24.67%), NT (22.15%) and RS (20.18%), respectively. The mean SOCD values for the 0–300 profiles were as follows: NT (5.38 kg m^−2^) is followed by ZX (4.63 kg m^−2^) and RS (4.39 kg m^−2^).

Figure 4(d–f) shows the variation in ZX, NT, and RS as well as the variation in SICD across various soil profiles. Overall, the SICD in the 0–300 cm soil layer did not significantly alter with soil profile. There was no statistically significant difference in SICD between ZX (6.76 kg m^−2^), NT (7.08 kg m^−2^), and RS (7.64 kg m^−2^) in the 0–100 cm soil depth range. The percentage of SICD in the 0–100 cm soil layer over the 0–300 cm soil depth was ZX (29.97%), NT (31.07%), and RS (29.11%), respectively. Differences in SICD at 100–200 cm soil depth were not significant between NT (7.88 kg m^−2^) and ZX (8.26 kg m^−2^), but were significant between RS (9.78 kg m^−2^). The percentage of SICD at 100–200 cm soil depth in the 0–300 cm soil layer was ZX (36.61%), NT (34.57%), and RS (37.24%), respectively. Within the 200–300 cm soil depth, SICD did not differ significantly between NT (7.84 kg m^−2^) and ZX (7.54 kg m^−2^), while it differed significantly with RS (8.84 kg m^−2^). The percentage of SICD at 200–300 cm soil depth in the 0–300 cm soil layer was ZX (33.41%), NT (34.36%), and RS (33.65%), respectively. The mean SICD for the entire 0–300 cm soil layer were, in descending order RS (8.76 kg m^−2^), NT (7.61 kg m^−2^), and ZX (7.52 kg m^−2^). Since the deep soil carbon density makes up more than 50% of the shallow soil, regional carbon sequestration will be greatly understated if just the shallow soil carbon density in the 0–100 cm soil layer is taken into account when estimating soil carbon density.

### Variation of SICD, SOCD, and STCD along the precipitation gradient in various soil strata

**Fig. 5:**
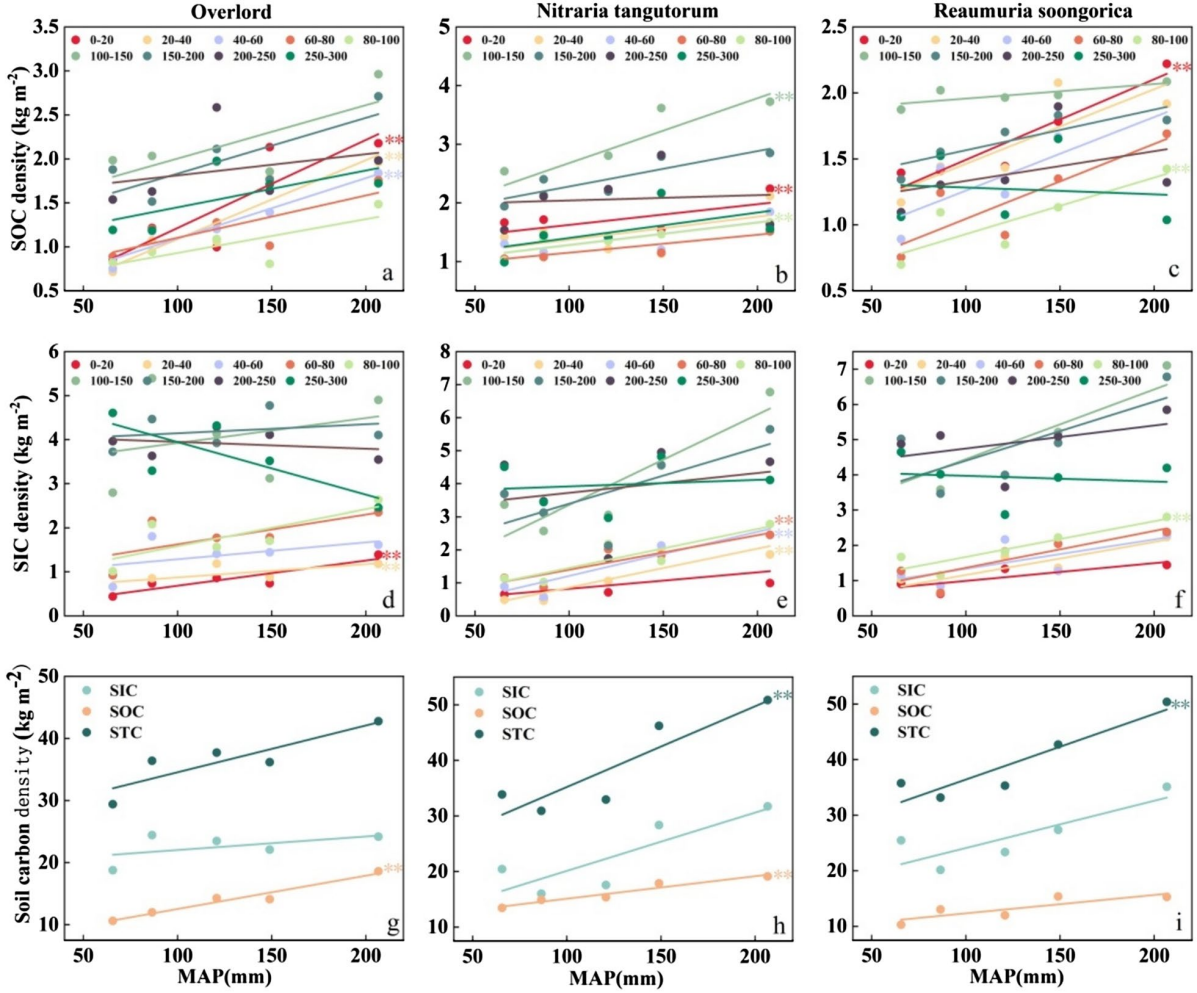
Variation of SICD, SOCD, and STCD along the precipitation gradients in various soil strata. **Indicates significance of linear regression at the 0.05 level. **a**, **b**, **c** denote the linear relationship between SOC density and precipitation for ZX, NT, and RS, respectively, **d**, **e**, **f** denote the linear relationship between SIC density and precipitation for ZX, NT, and RS, respectively, and **g**, **h**, **i** denote the linear relationship between total soil carbon density and precipitation for ZX, NT, and RS, respectively

The association between SOC, SIC, and STC concentrations and MAP in each soil layer of various vegetation types was examined using linear regression analysis (Fig. 5). As shown in Fig. 5 (a–c), ZX, NT, and RS found a significant positive correlation between SOCD and MAP in the surface 0–20 cm of the soil. ZX showed a significant positive correlation between SOCD and MAP at 0–60 cm soil depth. In the 80–150 cm soil depth, NT demonstrated a substantial positive link between SOCD and MAP. In the 80–100 cm soil depth, RS demonstrated a substantial positive association between SOC density and MAP. In other soil depths, SOCD and MAP did not significantly correlate according to ZX, NT, and RS.

The association between SICD and MAP is depicted in Fig. 5 (d–f). In the 0–40 cm soil layer, ZX revealed a sizable positive link between SIC density and MAP. In the 20–60 cm soil depth, NT found a considerable positive relationship between SICD and MAP. Only in the 80–100 cm soil depth did RS demonstrate a substantial positive association between SICD and MAP. Figure 5(g, h) depicts the association between total SIC, SOC, STC, and MAP in the 0–300 cm soil depth. The SOCD of ZX, STCD of NT, and SOCD of RS were all considerably and positively connected with MAP in the 0–300 cm soil depth. The three vegetation types’ SICD, SOCD, and STCD all rose with rising precipitation gradients, though not all of them significantly correlated with MAP.

### Factors influencing soil carbon density and their relative contributions

In Table 3, Person’s correlation analysis revealed that in the 0–200 cm soil depth, SOCD was strongly and positively linked with MAP and SMC. In the 0–300 cm depth, SOCD was substantially and positively correlated with soil type (ST), and in the 200–300 cm depth, SOCD was significantly and negatively correlated with pH. In the 0–300 cm soil depth, SOCD and MAT had a negative correlation that was not statistically significant. In the shallow 0–100 cm, SICD was likewise significantly and positively linked with MAP and SMC [10]. In the deep 100–300 cm depth, SICD was strongly and favorably connected with pH and ST. In the 0–300 cm, SOCD and ST had a strong and positive correlation. According to the Person correlation analysis, MAP, SMC, pH, and ST are the key variables influencing SOCD and SICD, with the other variables having a relatively minor impact [11].

**Table 3:** Pearson correlation analysis between SOCD and SICD and environmental variables

Variables	Depths(cm)	PT	MAP	MAT	SMC	BD	pH	ST
SOCD	0–20	0.172	0.767**	−0.414	0.401**	0.457*	0.325	0.536*
	20–40	0.291	0.751**	−0.365	0.370**	0.334	0.343	0.421*
	40–60	0.17	0.850**	−0.398	0.312**	0.358	0.593*	0.432
	60–80	0.052	0.807**	−0.462	0.332*	0.292	0.206	0.571*
	80–100	0.014	0.69**	−0.369	0.411**	0.145	0.387	0.668**
	100–150	0.118	0.498*	−0.284	0.658**	0.034	0.014	0.647**
	150–200	0.313	0.557	−0.312	0.42*	0.317	0.137	0.545*
	200–250	0.316	0.194	−0.249	0.368	0.107	-0.465*	0.581*
	250–300	0.338	0.381	−0.262	0.071	0.201	-0.142*	0.479*
SICD	0–20	0.296	0.653**	0.424	0.518*	0.017	0.553	0.376
	20–40	0.346	0.75**	0.368	0.68*	0.031	0.391	0.545*
	40–60	0.117	0.723**	0.421	0.509*	0.139	0.417*	0.424*
	60–80	0.119	0.808*	0.368	0.411	0.159	0.396	0.504*
	80–100	0.096	0.846*	0.483	0.594*	0.033	0.552*	0.408**
	100–150	0.272	0.658	0.361	0.479	0.333*	0.527*	0.598*
	150–200	0.244	0.556	0.219	−0.26*	0.066	0.719*	0.505*
	200–250	0.435	0.192	0.367	−0.302	0.104	0.652**	0.661**
	250–300	0.172	0.274	0.234	−0.205	0.128	0.649**	0.704**

^*^denotes significant correlation at the P < 0.05 probability level. **indicates significant correlation at the P < 0.01 probability level. Plant type (*PT*), multi-annual average precipitation (*MAP*), multi-annual average temperature (*MAT*), soil moisture content (*SMC*) Soil bulk density (*BD*), Soil type (*ST*)

**Fig. 6:**
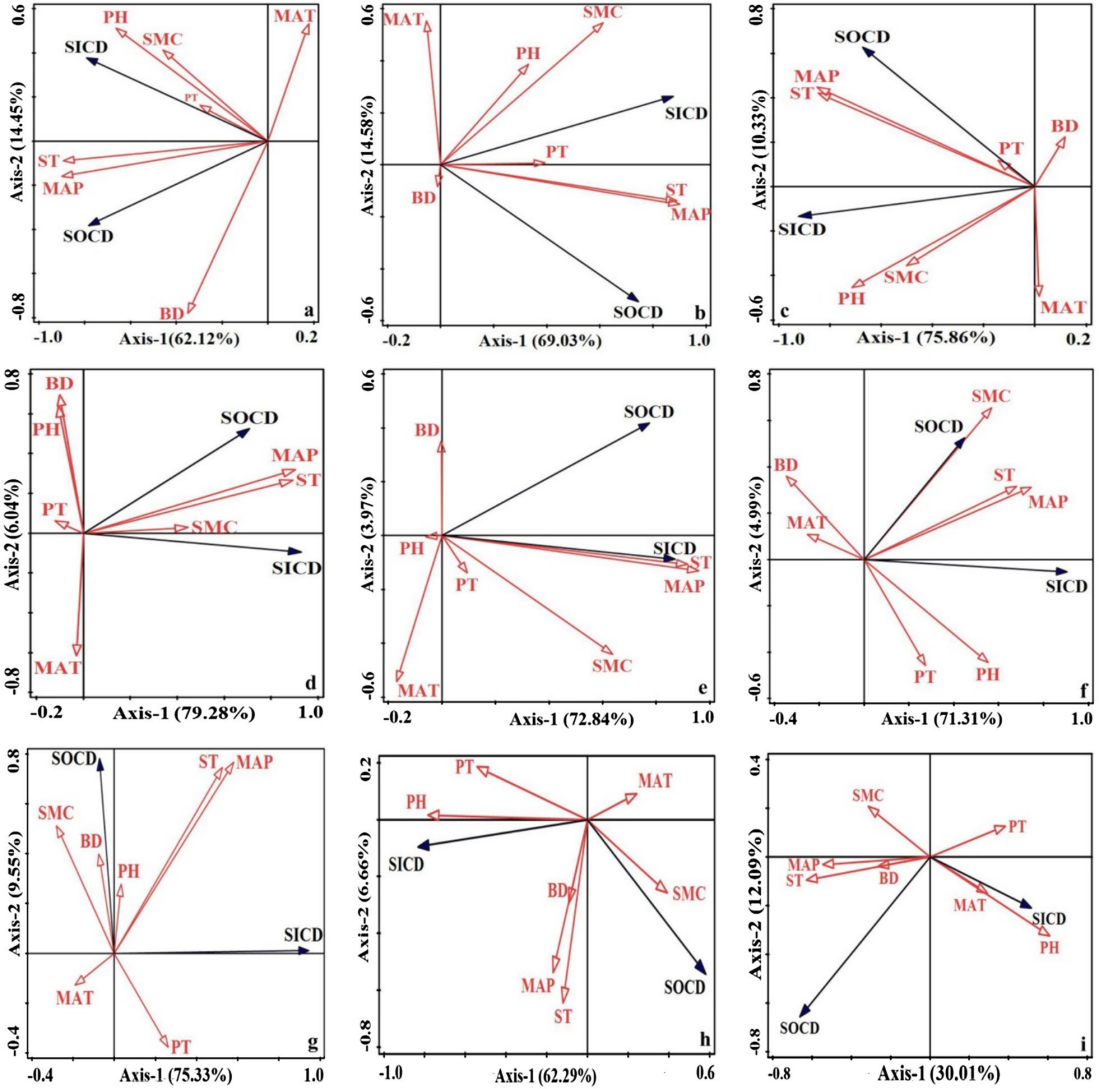
RDA two-dimensional ordination plots of the correlation of SIC and SOC with environmental factors for different soil layers with ordinal numbers (**a–i)** in the order of soil layers 0–20, 20–40, 250–300 cm. The abbreviations in the figure are respectively: plant type (*PT*), multi-annual average precipitation (MAP), multi-annual average temperature (*MAT*), soil moisture content (*SMC*) soil bulk density (*BD*), soil type *(ST*)

The two-dimensional ordination diagram for SOCD and SICD correlations with each physicochemical element is shown in Fig. 6 as a result of RDA ranking. The size of the correlation is shown in Fig. 6 by the length and direction of the arrows; the longer the arrow, the higher the correlation, and the greater the cosine value between the arrow angles, the higher the correlation [21]. As shown in Fig. 6, the amount of explanation of SOCD and SICD by seven environmental factors gathered in the first two axes reached 76.57%, 83.61%, 86.19%, 85.32%, 76.81%, and 76.3%, respectively, 84.88%, 68.95%, and 42.1% in the 0–20cm, 20–40cm, and 250–300cm soil layers. The impacts of MAP, ST, SD, SMC, and pH on SOCD and SICD were stronger in the 0–20 cm soil depth [28]. MAP, SMC, and ST had a stronger impact on SOCD and SICD in the 20–40 cm soil depth. In the 40–60cm depth, the impacts of MAP, SMC, pH, and ST on SOCD and SICD were more pronounced. In the 60–100cm depth, the impacts of MAP, MAT, SMC, and ST on SOCD and SICD were stronger. In the 100–150cm depth, the impacts of MAP, SMC, pH, and ST on SOCD and SICD were stronger [31]. In the 150–250cm depth, the impacts of MAP, SMC, pH, and ST on SOCD and SICD were stronger in the 200–250 cm soil layer. In the 250–300cm depth, the impacts of MAP, pH, and ST on SOCD and SICD were stronger [33].

**Fig. 7:**
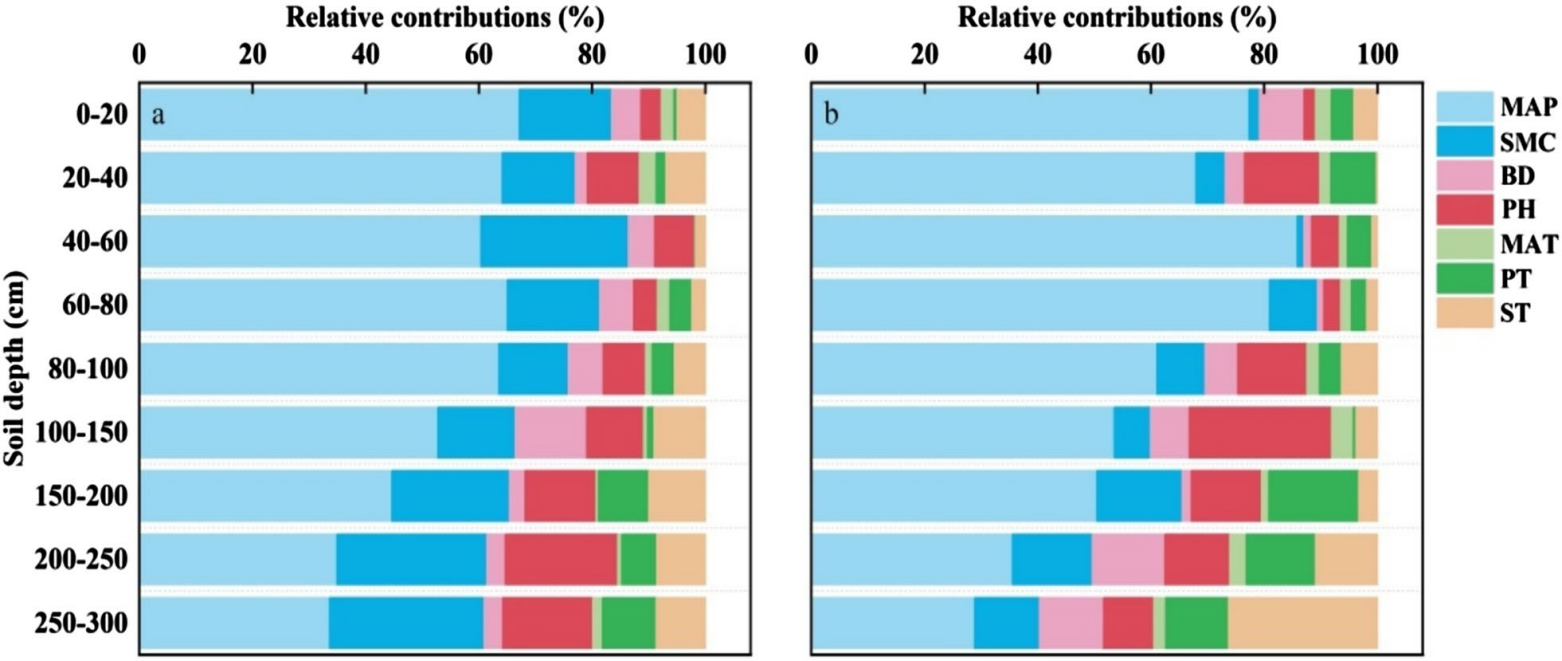
Relative contribution of environmental factors to (**a**) SOC and (**b**) SIC density at each depths

The respective contribution of the primary environmental elements to SOCD and SICD at each depth is calculated in Fig. 7. The average variance in SOCD was described by MAP, pH, SMC, ST, BD, PT, and MAT, while the average variance in SICD was explained by 53.97%, 19.13%, 10.01%, 6.44%, and 5.08%, respectively, and 3.99% and 1.35%.

## Discussion

### Patterns of spatial distribution of SOC over a precipitation gradient

Several investigations have shown that fluctuations in SOC content are mostly caused by environmental factors such as precipitation and temperature [49, 62, 68]. This study’s redundancy analysis revealed that precipitation was the primary cause of the fluctuation in SOC content. At these five sites on the Alxa Plateau, we discovered that SOC content rose with MAP in this study, showing that potential SOC buildup should be greater in wetter places than in dry areas. Previous research on the fluctuation of SOC content with precipitation gradient in the Inner Mongolia Plateau, China [18], and northwestern territories, Iran [61] can also be used to corroborate the conclusions of this study. Because soil moisture effectiveness is typically a significant constraint for plant output, particularly in arid desert locations, SOC content increased as MAP increased [35, 97, 104]. This is also illustrated by the fact that SMC contributed to SOC content to a lesser extent than MAP in this study, and the contribution of SMC to SOC content became progressively more prominent with soil depth change [37]. According to Huang et al. [30] and Hong et al. [26], increased precipitation typically results in increased soil moisture, which boosts plant productivity and litter inputs. Arid soils also have an impact on how water infiltration moves organic matter from shallow to deep soils. Soil moisture effectiveness may also be a limiting element in the organic carbon generation from microbial degradation of plant litter in deep soils [96]. In dry and semi-arid terrestrial ecosystems, MAP is also a significant limiting factor for productivity and the sustainability of development [2, 7, 9]. In this study, the link between SOCD and MAP was quite favorable. At every soil depth, as the gradient of precipitation grew, SOCD grew as well. According to Banning et al. [3] and Iglesias et al. [31], more litter is produced as a result of increased aboveground biomass due to increased precipitation, which is a key source of soil nutrients through microbial breakdown processes [4].

For the reason of the efficient buildup of litter inputs and microbial decomposition activities of above and below-ground biomass, shallow soil (0–100 cm) had a stronger association between SOC and MAP than deep soil [19, 56, 91]. When precipitation rose, soil moisture recharge conditions allowed for the full growth and accumulation of above and below-ground biomass, which encouraged litter input and microbial activity. For instance, it was discovered that an average of 95% of woody vegetation at six paired sites had roots depths > 200 cm across a rainfall gradient in the southwest of the United States, with a corresponding increase in biomass with the rainfall gradient and a gradually decreasing root density with soil depth [84]. Because root biomass is assumed to be the primary source of soil nutrients, SOC decreases with soil depth [19, 21, 37, 53, 83, 99]. By using Person correlation analysis, it was also discovered that SOC content was negatively linked with MAT. This might be because hotter temperatures hasten evapotranspiration, which decreases soil moisture availability and plant yield [73–75, 79, 86]. Through its impact on microbial breakdown, temperature can regulate fluctuations in SOC inventory [50]. At our sampling sites, MAT’s influence on variations in SOC content was less noticeable. The non-significant variances in MAT between the five study sites, which ranged from 7.9 to 9.1 ℃, may help to explain the phenomena. The upgrading of SOC distribution patterns from the stand scale to the regional scale is supported by our results, which subtly highlight the significance of taking into account both soil depth and rainfall when measuring SOC density.

### Patterns of spatial distribution of SIC over a precipitation gradient

Raheb et al. [61] looked at how MAP affected SIC pools in dry, semi-arid, and sub-humid environments. With the increase in MAP, total SICD increased from 3.75 kg m^−2^ under arid and semi-arid conditions to 11.32 kg m^−2^ under sub-humid dry conditions, respectively. The highest SIC/SOC ratio was found in the arid desert region, where soils had lower SOC contents. The significant impact of precipitation on SIC storage is indicated by the high value of this ratio compared to SOC [38]. Arid soils are enriched with pedogenic inorganic carbon (PIC) thanks to precipitation ratios during high evaporation circumstances that prevent carbonate breakdown and leaching. The development and accretion of rocky calcareous and calcareous horizons in arid regions with limited effective precipitation is facilitated by an increase in SIC content, according to a study by Wu et al. [87]. The potential for SIC accumulation is greater in arid locations with poor effective precipitation than in humid regions with rich effective precipitation, according to this study’s finding that SICD showed the same increasing tendency with precipitation gradient [38]. For instance, SIC reservoirs are more prevalent in regions with annual precipitation below 500 mm [40, 59, 60]. According to [54, 55], there are 84% of China’s total SIC reservoirs in areas with MAP is less than 500 mm [40]. Seasonal droughts result in decreased root activity and soil moisture, and seasonal dry periods are suitable for carbonate precipitation [23, 64, 103]. The average annual precipitation of the Alxa Plateau is mainly distributed in July–September, with little precipitation in the other periods, thus allowing a longer period of carbonate precipitation [53].

The overall distribution of SIC across the soil profile similarly showed a pattern of smaller in the lower layers and greater in the deeper layers. The lower soil pH and higher SOC in the topsoil can be linked to this pattern, where SOC decomposition can produce an acidic environment that encourages carbonate dissolution [70, 73–75, 79, 101]. Second, increased CO_2_ partial pressure and SMC could be the cause of a smaller SIC in the topsoil. This causes Eq. (3) to be shifted to the left and results in carbonate dissolution. In the deep soil profile, changes in soil inorganic carbon content were mostly impacted by root biomass, Ca^2+^, and HCO_3_^−^ and CO_2_ partial pressure. The root biomass of the deep soil grew in step with the rise in precipitation. Firstly, the input of root litter material increased, which stimulated the activity of soil fauna and microorganisms and accelerated the decomposition of litter material, and the unstable SOC was mineralized to produce more CO_2_, which drove Eq. (4) to carry on to the left, and was further dissolved in soil solution to form HCO_3_^−^, that was subsequently combined with the decomposed Ca^2+^ released from litter material combines and precipitates as CaCO_3_ [101]. Secondly, in extreme arid soil environments, PIC can be formed from the enrichment of unutilized excess Ca^2+^ by roots in the mucilaginous sheaths around root hairs, and the accumulation of large amounts of HCO_3_^−^ in the mucilaginous sheaths as a result of respiration, which makes the mucilaginous sheaths provide a unique environment for the combination of Ca^2+^and HCO_3_^−^, and in both cases, the action of these two, PIC development in deep soils is encouraged [41, 58, 89]. Finally, The possibility that deeper soils have higher SIC is connected to the rainy season’s carbonate leaching from topsoil to deeper soil [54, 55, 90, 100].

3 $${\text{Ca}}^{2 + } + 2{\text{HCO}}_{3}^{ - } \leftrightarrow {\text{CaCO}}_{3} + {\text{H}}_{2} {\text{O}} + {\text{CO}}_{2}$$

4 $${\text{CaCO}}_{3} + 2{\text{H}}^{ + } \leftrightarrow {\text{HCO}}_{3}^{ - } + {\text{H}}^{ + } + {\text{Ca}}^{2 + } \leftrightarrow {\text{H}}_{2} {\text{O}} + {\text{CO}}_{2} + {\text{Ca}}^{2 + }$$

### Correlation between SIC and SOC

**Fig. 8:**
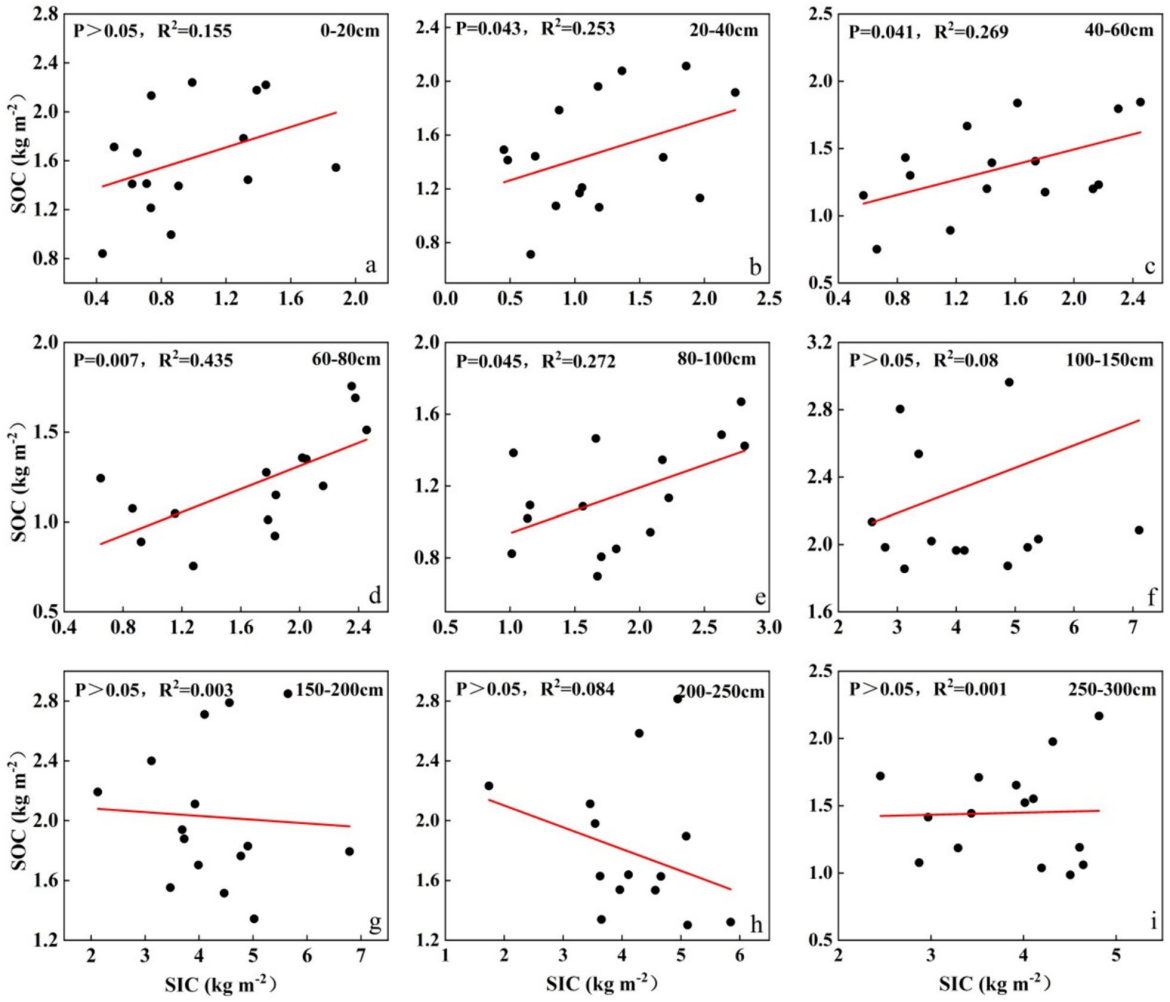
Correlation between SOCD and SICD

SIC and SOC relationships can be diverse, with positive [73–75, 79], negative [101] or uncorrelated relationships [51]. For instance, in China, topsoil samples from the western Loess Plateau and the North China Plain revealed an inverse relationship between SIC and SOC [29, 95], while samples from the Badan jirin Desert in China and the Yanqing Basin in Xinjiang demonstrated that SIC and SOC had a favorable connection [69, 78]. In arid ecosystems, the link between SIC and SOC is maintained in large part by soil pH [64, 74]. More CO_2_ will be released into the soil as a result of microbial decomposition of SOC and plant root respiration, making the soil more acidic [74]. A negative link between the surface SIC and SOC results from carbonate dissolution being encouraged by low pH [20, 36]. In this work, as can be seen from Fig. 8, SIC and SOC within the top 100 cm depth, on the other hand, showed an intense positive connection. Because the soils of the five sites in this study were alkaline soils, the soil parent material was brown desert soil type or calcareous soil type, with high pH and abundant Ca^2+^, Mg^2+^ and other ions, carbonic acid was formed after the decomposition of SOC to form CO_2_, and reacted with Ca^2+^, Mg^2+^ and other ions to form carbonate-like substances stored in the soil [13, 47]. In addition, alkaline soils are less likely to cause solubilization of SIC, thus SIC and SOC have a significant favorable association [6, 27]. Differences in SIC-SOC correlations reflect the fact that SIC and SOC may behave differently in response to changes in environmental conditions [64]. For example, SOC was less stable at high soil pH, and soil salinity inhibited the effective accumulation of SOC [65]. On the other hand, it increased the concentrations of Mg^2+^ and Ca^2+^ in the soil, which promoted the accumulation of SIC, resulting in a bad correlation between SIC and SOC [15, 48, 85]. Whereas, under the conditions of high SMC and pH, the decomposition of SOC and leaching of SIC increased, thus indicating a positive relationship between SIC and SOC [20, 71, 98].

Since the research on the interrelationship between SIC and SOC is still limited, the results of different land use or the same land use in different regions indicate that the interaction between SIC and SOC is very sophisticated, which may be a reflection of the spatial and temporal superposition of the various processes or reactions that lead to the formation of carbonates. In addition, both the SOC/SIC values and the correlation analysis of SOC and SIC contents can only speculate or support the interrelationship between SOC and SIC contents from the statistical results. Still, they cannot directly explain the transformation process between SOC and SIC. Therefore, relevant studies should be strengthened in the future to clarify the interrelationship between SIC and SOC contents under different environmental factors, and to enhance the mechanistic study of the soil carbonate transformation process with the help of modeling and isotope methods, to have a solid scientific foundation for fairly assessing how much SIC contributes to the global soil carbon balance.

### Unexplained changes in SIC and SOC accumulation

In this work, SIC and SOC fluctuations in the 0–300 cm soil depth were analyzed, instead of using straightforward qualitative assertions or correlation analyses, the majority of the variations in soil carbon pools were successfully explained. This result provides a key theory in terms of better explaining the causes of SIC and SOC changes. Conversely, a fraction of the variations in soil carbon buildup are still not well understood. Lack of information regarding soil clay, chalk, and sand particles related to soil texture may be to blame for the inadequate explanation for some of the SOC changes. One of the key elements influencing the difference in SOC content is the proportion of sand, silt, and clay in soil particles, especially in deep soils. Additionally, soil aggregates provide physical protection against the formation of SOC content [67, 88]. A small amount of organic matter inputs from above and below-ground apoplastic material also helps to boost SOC content [12, 19]. Most soil microorganisms are heterotrophic, using soil organic matter as a source of energy and carbon, and SOC accumulation is also correlated with root apoplastic decomposition and the intensity of microbial activity. Differences in the characterization of soil microbial communities may also explain some of the SOC decomposition [32]. As a result, a dearth of information about elements like plant litter, root biomass, and microbial communities may be to blame for some of the inexplicable changes in SOC.

The the inadequate explanation of some of the SIC variations can be a result of a lack of information on soil conductivity, soil texture, and soil salinity. Soil texture and structure control, among other things, soil permeability, and water dynamics, and therefore indirectly influence the depth of PIC accumulation [11, 44, 98, 100]. The geographical distribution of Ca^2+^ and Mg^2+^ plasma content in the soil is influenced by soil salinity and conductivity, which has an impact on SIC formation and transmission. The absence of information on the physicochemical characteristics of the soil in the deep inter-root layer of the soil may be one of the reasons why there is also a portion of SIC buildup that cannot be explained. When there are substantial, live roots, carbonate dissolution is greatly increased. This is due to the release of H^+^ and carboxylic acids by roots [1, 22], which makes the inter-root CO_2_ concentration 100 fold higher than in the atmosphere, and the local pH of inter-root soils two units lower than that of non-inter-root soils, leading to an increase in the dissolution of carbonate near roots. The absence of information on soil microorganisms could be the second explanation. Fungi and bacteria found in soil play a major role in the development of PIC. Bacteria can quickly accumulate visible carbonate if there are Ca^2+^ and Mg^2+^ in the fluid [8, 57, 102].

## Conclusions

This study investigated the changes in SOC and SIC of three vegetation types in the 0–300cm soil profile and their variation with precipitation gradients in five regions from western to eastern Alxa region. Firstly, the SICD and SOCD of these five regions increase with the increase of MAP. Among these three types of vegetation, the SOC content and density are in the order of NT > RS > ZX, and the SIC content and density are in the order of RS > NT > ZX. Secondly, Pearson and RDA analyses indicate that MAP, SMC, pH, and ST have a significant impact on soil carbon density in arid desert areas. This study indirectly emphasizes the importance of considering both soil depth and rainfall when evaluating soil carbon density, which can support the upgrading of soil carbon density distribution patterns from stand scale to regional scale. In order to gain a deeper understanding of the impact mechanism of soil carbon density, subsequent research should also investigate relevant information such as soil texture, vegetation roots, and biomass, in order to gain a more comprehensive understanding of changes in soil carbon density.

## Data Availability

Data will be provided by the corresponding author upon request.

## References

[1] Andrews JA, Schlesinger WH (2001). Soil CO2 dynamics, acidification, and chemical weathering in a temperate forest with experimental CO2 enrichment. Global Biogeochem Cycle.

[2] Aranibar JN, Otter L, Macko SA, Feral C, Epstein HE, Dowty PR, Eckardt F, Shugart HH, Swap RJ (2004). Nitrogen cycling in the soil-plant system along a precipitation gradient in the Kalahari sands. Glob Change Biol.

[3] Banning NC, Grant CD, Jones DL, Murphy DV (2008). Recovery of soil organic matter, organic matter turnover and nitrogen cycling in a post-mining forest rehabilitation chronosequence. Soil Biol Biochem.

[4] Barcena TG, Kiaer LP, Vesterdal L, Stefansdottir HM, Gundersen P, Sigurdsson BD (2014). Soil carbon stock change following afforestation in northern Europe: a meta-analysis. Glob Change Biol.

[5] Batjes NH (1996). Total carbon and nitrogen in the soils of the world. Eur J Soil Sci.

[6] Batjes NH (2014). Total carbon and nitrogen in the soils of the world. Eur J Soil Sci.

[7] Bradford MA, Wieder WR, Bonan GB, Fierer N, Raymond PA, Crowther TW (2016). Managing uncertainty in soil carbon feedbacks to climate change. Nat Clim Chang.

[8] Bughio MA, Wang P, Meng F, Qing C, Kuzyakov Y, Wang X, Junejo SA (2016). Neoformation of pedogenic carbonates by irrigation and fertilization and their contribution to carbon sequestration in soil. Geoderma.

[9] Campo J, Merino A (2016). Variations in soil carbon sequestration and their determinants along a precipitation gradient in seasonally dry tropical forest ecosystems. Glob Change Biol.

[10] Carvalhais N, Forkel M, Khomik M, Bellarby J, Jung M, Migliavacca M, Μu M, Saatchi S, Santoro M, Thurner M, Weber U, Ahrens B, Beer C, Cescatti A, Randerson JT, Reichstein M (2014). Global covariation of carbon turnover times with climate in terrestrial ecosystems. Nature.

[11] Chadwick OA, Sowers JM, Amundson RG (1989). Morphology of calcite crystals in clast coatings from 4 soils in the mojave desert region. Soil Sci Soc Am J.

[12] Chang R, Fu B, Liu G, Wang S, Yao X (2012). The effects of afforestation on soil organic and inorganic carbon: a case study of the loess plateau of China. CATENA.

[13] Chaparro-Acuña SP, Becerra-Jiménez ML, Martínez-Zambrano JJ, Rojas-Sarmiento HA (2018). Soil bacteria that precipitate calcium carbonate mechanism and applications of the process. Acta Agronómica.

[14] Chen Z, Wei X, Ni X, Wu F, Liao S (2023). Changing precipitation effect on forest soil carbon dynamics is driven by different attributes between dry and wet areas. Geoderma.

[15] Demoling F, Figueroa D, Bååth E (2007). Comparison of factors limiting bacterial growth in different soils. Soil Biol Biochem.

[16] Díaz-Hernández JL (2010). Is soil carbon storage underestimated?. Chemosphere.

[17] Eswaran H, Reich PF, Kimble JM, Beinroth FH, Padmanabhan E, Moncharoen P, Lal R, Kimble JM, Eswaran H, Stewart BA (2000). Global carbon stocks. “Global Climate Change And Pedogenic Carbonates” Workshop on global climate change and pedogenic carbonates.

[18] Evans SE, Burke IC, Lauenroth WK (2011). Controls on soil organic carbon and nitrogen in in Inner Mongolia, China: a cross-continental comparison of comparison of temperate grasslands. Global Biogeochem Cycle.

[19] Feng J, He K, Zhang Q, Han M, Zhu B (2022). Changes in plant inputs alter soil carbon and microbial communities in forest ecosystems. Glob Change Biol.

[20] Gao Y, Dang P, Zhao Q, Liu J, Liu J (2018). Effects of vegetation rehabilitation on soil organic and inorganic carbon stocks in the Mu Us Desert, northwest China. Land Degrad Dev.

[21] Giardina CP, Ryan MG, Binkley D, Fownes JH (2003). Primary production and carbon allocation in relation to nutrient supply in a tropical experimental forest. Glob Change Biol.

[22] Gocke M, Pustovoytov K, Kuzyakov Y (2011). Carbonate recrystallization in root-free soil and rhizosphere of *Triticum aestivum* and *Lolium perenne *estimated by C-14 labeling. Biogeochemistry.

[23] Guasconi D, Manzoni S, Hugelius G (2023). Climate-dependent responses of root and shoot biomass to drought duration and intensity in grasslands-a meta-analysis. Sci Total Environ.

[24] Han X (2018). Shape-preserving piecewise rational interpolation with higher order continuity. Appl Math Comput.

[25] Hirmas DR, Amrhein C, Graham RC (2010). Spatial and process-based modeling of soil inorganic carbon storage in an arid piedmont. Geoderma.

[26] Hong J, Wang X, Wu J (2015). Effects of soil fertility on the NP stoichiometry of herbaceous plants on a nutrient-limited alpine steppe on the northern Tibetan Plateau. Plant Soil.

[27] Hong S, Chen A (2022). Contrasting responses of soil inorganic carbon to afforestation in acidic versus alkaline soils. Global Biogeochem Cycle.

[28] Houghton RA, Davidson EA, Woodwell GM (1998). Missing sinks, feedbacks, and understanding the role of terrestrial ecosystems in the global carbon balance. Global Biogeochem Cycle.

[29] Huang B, Wang J, Jin H, Xu S (2006). Effects of long-term application fertilizer on carbon storage in calcareous meadow soil. J Agro-environ sci.

[30] Huang K, Zhang Y, Zhu J, Liu Y, Zu J, Zhang J (2016). The influences of climate change and human activities on vegetation dynamics in the Qinghai-Tibet plateau. Remote Sensing.

[31] Iglesias MDR, Barchuk A, Grilli MP (2012). Carbon storage, community structure and canopy cover: A comparison along a precipitation gradient. For Ecol Manage.

[32] Jackson LE, Calderon FJ, Steenwerth KL, Scow KM, Rolston DE (2003). Responses of soil microbial processes and community structure to tillage events and implications for soil quality. Geoderma.

[33] Jeong J, Kim C, Lee K, Bolan NS, Naidu R (2013). Carbon storage and soil CO2 efflux rates at varying degrees of damage from pine wilt disease in red pine stands. Sci Total Environ.

[34] Jia X, Yang Y, Zhang C, Shao M, Huang L (2017). A state-space analysis of soil organic carbon in China’s loess plateau. Land Degrad Dev.

[35] Jobbagy EG, Jackson RB (2000). The vertical distribution of soil organic carbon and its relation to climate and vegetation. Ecol Appl.

[36] Kolosz BW, Sohi SP, Manning DAC (2019). CASPER: a modelling framework to link mineral carbonation with the turnover of organic matter in soil. Comput Geosci.

[37] Laganiere J, Angers DA, Pare D (2010). Carbon accumulation in agricultural soils after afforestation: a meta-analysis. Glob Change Biol.

[38] Lal R (2004). Soil carbon sequestration to mitigate climate change. Geoderma.

[39] Lal R (2013). Soil carbon management and climate change. Carbon MANAGEMENT.

[40] Lal R, Kimble JM, Lal R, Kimble JM, Eswaran H, Stewart BA (2000). Pedogenic carbonates and the global carbon cycle. Global Climate Change And Pedogenic Carbonates”.

[41] Lalitha M, Dharumarajan S, Khandal S, Koyal A, Parvathy S, Kalaiselvi B, Kumar KSA, Hegde R (2022). Vertical distribution of soil organic and inorganic carbon under silvipastoral system in a dry semiarid agro-ecological region, Tamil Nadu, India. Range Manage Agroforestry.

[42] Lan ZL, Zhao Y, Zhang JG, Jiao R, Khan MN, Sial TA, Si BC (2021). Long-term vegetation restoration increases deep soil carbon storage in the northern loess plateau. Sci Report.

[43] Li M, Zhang X, Pang G, Han F (2013). The estimation of soil organic carbon distribution and storage in a small catchment area of the loess plateau. CATENA.

[44] Liang, S., Tang, J., and IOP 2018. Dynamic variation characterization of soil organic and inorganic carbon of saline-alkali paddy fields and the influence factors. *In* “2018 International Conference on air pollution and environmental engineering (APEE 2018)”, International Conference on Air Pollution and Environmental Engineering (APEE).

[45] Liu J, Wu P, Zhao Z, Gao Y (2022). Afforestation on cropland promotes pedogenic inorganic carbon accumulation in deep soil layers on the Chinese loess plateau. Plant Soil.

[46] Liu S, Bond-Lamberty B, Boysen LR, Ford JD, Fox A, Gallo K, Hatfield J, Henebry GM, Huntington TG, Liu Z, Loveland TR, Norby RJ, Sohl T, Steiner AL, Yuan W, Zhang Z, Zhao S (2017). Grand challenges in understanding the interplay of climate and land changes. Earth Interact.

[47] Liu X, Monger HC, Whitford WG (2007). Calcium carbonate in termite galleries—biomineralization or upward transport?. Biogeochemistry.

[48] Liu Z, Li J, Zhang Y, Gong H, Hou R, Sun Z, Ouyang Z (2023). Soil microbes from saline-alkali farmland can form carbonate precipitates. Agronomy-Basel.

[49] Liu Z, Shao MA, Wang Y (2011). Effect of environmental factors on regional soil organic carbon stocks across the loess plateau region, China. Agr Ecosyst Environ.

[50] Lu M, Zhou X, Yang Q, Li H, Luo Y, Fang C, Chen J, Yang X, Li B (2013). Responses of ecosystem carbon cycle to experimental warming: a meta-analysis. Ecology.

[51] Lu T, Wang X, Xu M, Yu Z, Luo Y, Smith P (2020). Dynamics of pedogenic carbonate in the cropland of the north China plain: influences of intensive cropping and salinization. Agr Ecosyst Environ.

[52] Mathieu JA, Hatte C, Balesdent J, Parent E (2015). Deep soil carbon dynamics are driven more by soil type than by climate: a worldwide meta-analysis of radiocarbon profiles. Glob Change Biol.

[53] Mergel A, Timchenko A, Kudeyarov V, Box JE (1998). Role of plant root exudates in soil carbon and nitrogen transformation. “Root demographics and their efficiencies in sustainable agriculture, grasslands and forest ecosystems”, 5th symposium of the international-society-of-root-research.

[54] Mi N, Wang SQ, Liu JY, Yu GR, Zhang WJ, Jobbaagy E (2008). Soil inorganic carbon storage pattern in China. Glob Change Biol.

[55] Mi N, Wang S, Liu J, Yu G, Zhang W, Jobbagy E (2008). Soil inorganic carbon storage pattern in China. Glob Change Biol.

[56] Miao R, Ma J, Liu Y, Liu Y, Yang Z, Guo M (2019). Variability of aboveground litter inputs alters soil carbon and nitrogen in a coniferous-broadleaf mixed forest of central China. Forests.

[57] Monger HC, Daugherty LA, Lindemann WC, Liddell CM (1991). Microbial precipitation of pedogenic calcite. Geology.

[58] Monger HC, Kraimer RA, Khresat S, Cole DR, Wang X, Wang J (2015). Sequestration of inorganic carbon in soil and groundwater. Geology.

[59] Naorem A, Jayaraman S, Dalal RC, Patra A, Rao CS, Lal R (2022). Soil inorganic carbon as a potential sink in carbon storage in dryland soils-a review. Agriculture-Basel.

[60] Pfeiffer M, Padarian J, Vega MP (2023). Soil inorganic carbon distribution, stocks and environmental thresholds along a major climatic gradient. Geoderma.

[61] Raheb A, Heidari A, Mahmoodi S (2017). Organic and inorganic carbon storage in soils along an arid to dry sub-humid climosequence in northwest of Iran. CATENA.

[62] Ren Z, Li C, Fu B, Wang S, Stringer LC (2024). Effects of aridification on soil total carbon pools in China’s drylands. Global Change Biol.

[63] Schindlbacher A, Beck K, Holzheu S, Borken W (2019). Inorganic carbon leaching from a warmed and irrigated carbonate forest soil. Front Forests Global Change.

[64] Schlesinger WH (1982). Carbon storage in the caliche of arid soils–a case-study from Arizona. Soil Sci.

[65] Schlesinger WH (1985). The formation of caliche in soils of the mojave-desert, California. Geochim Cosmochim Acta.

[66] Selhorst A, Lal R (2012). Effects of climate and soil properties on US home lawn soil organic carbon concentration and pool. Environ Manage.

[67] Six J, Conant RT, Paul EA, Paustian K (2002). Stabilization mechanisms of soil organic matter: implications for C-saturation of soils. Plant Soil.

[68] Spohn M, Bagchi S, Biederman LA, Borer ET, Brathen KA, Bugalho MN, Caldeira MC, Catford JA, Collins SL, Eisenhauer N, Hagenah N, Haider S, Hautier Y, Knops JMH, Koerner SE, Laanisto L, Lekberg Y, Martina JP, Martinson H, Mcculley RL, Peri PL, Macek P, Power SA, Risch AC, Roscher C, Seabloom EW, Stevens C, Veen GFC, Virtanen R, Yahdjian L (2023). The positive effect of plant diversity on soil carbon depends on climate. Nat Commun.

[69] Su YZ, Wang XF, Yang R, Lee J (2010). Effects of sandy desertified land rehabilitation on soil carbon sequestration and aggregation in an arid region in China. J Environ Manage.

[70] Sun Z, Meng F, Zhu B (2023). Influencing factors and partitioning methods of carbonate contribution to CO_2 emissions from calcareous soils. Soil Ecol Lett.

[71] Tong LS, Fang NF, Xiao HB, Shi ZH (2020). Sediment deposition changes the relationship between soil organic and inorganic carbon: evidence from the Chinese loess plateau. Agriculture Ecosyst Environ.

[72] Tuo D, Gao G, Chang R, Li Z, Ma Y, Wang S, Wang C, Fu B (2018). Effects of revegetation and precipitation gradient on soil carbon and nitrogen variations in deep profiles on the loess plateau of China. Sci Total Environ.

[73] Wang D, Liu Y, Wu G, Ding L, Yang Z, Hao H (2015). Effect of rest-grazing management on soil water and carbon storage in an arid grassland (China). J Hydrol.

[74] Wang JP, Wang XJ, Zhang J, Zhao CY (2015). Soil organic and inorganic carbon and stable carbon isotopes in the Yanqi basin of northwestern China. Eur J Soil Sci.

[75] Wang J, Cao H, Liao Y, Liu W, Tan L, Tang Y, Chen J, Xu X, Li H, Luo C, Liu C, Ries Merikangas K, Calhoun V, Tang J, Shugart YY, Chen X (2015). Three dysconnectivity patterns in treatment-resistant schizophrenia patients and their unaffected siblings. NeuroImage Clinical.

[76] Wang T, Piao S (2023). Estimate of terrestrial carbon balance over the tibetan plateau: progresses, challenges and perspectives. Quat Sci.

[77] Wang W, Chen X, Zheng H, Yu R, Qian J, Zhang Y, Yu J (2016). Soil CO2 uptake in deserts and its implications to the groundwater environment. Water.

[78] Wang XJ, Xu MG, Wang JP, Zhang WJ, Yang XY, Huang SM, Liu H (2014). Fertilization enhancing carbon sequestration as carbonate in arid cropland: assessments of long-term experiments in northern China. Plant Soil.

[79] Wang X, Wang J, Xu M, Zhang W, Fan T, Zhang J (2015). Carbon accumulation in arid croplands of northwest China: pedogenic carbonate exceeding organic carbon. Sci Report.

[80] Wang YQ, Han XW, Jin Z, Zhang CC, Fang LC (2016). Soil Organic Carbon Stocks in Deep Soils at a Watershed Scale on the Chinese Loess Plateau. Soil Sci Soc Am J.

[81] Wang Y, Li Y, Ye X, Chu Y, Wang X (2010). Profile storage of organic/inorganic carbon in soil: from forest to desert. Sci Total Environ.

[82] Warren CJ, Saurette DD, Gillespie AW (2021). Soil organic carbon content: decreases partly attributed to dilution by increased depth of cultivation in southern Ontario. Can J Soil Sci.

[83] Wen L, Dong S, Li Y, Wang X, Li X, Shi J, Dong Q (2013). The impact of land degradation on the C pools in alpine grasslands of the Qinghai-Tibet Plateau. Plant Soil.

[84] Wiesmeier M, Spoerlein P, Geuss U, Hangen E, Haug S, Reischl A, Schilling B, von Luetzow M, Koegel-Knabner I (2012). Soil organic carbon stocks in southeast Germany (Bavaria) as affected by land use, soil type and sampling depth. Glob Change Biol.

[85] Wilsey B, Xu X, Polley HW, Hofmockel K, Hall SJ (2020). Lower soil carbon stocks in exotic vs. native grasslands are driven by carbonate losses. Ecology.

[86] Wu G, Ren G, Wang D, Shi Z, Warrington D (2013). Above and below-ground response to soil water change in an alpine wetland ecosystem on the Qinghai-Tibetan Plateau, China. J Hydrol.

[87] Wu H, Guo Z, Gao Q, Peng C (2009). Distribution of soil inorganic carbon storage and its changes due to agricultural land use activity in China. Agr Ecosyst Environ.

[88] Xu X, Shi Z, Li D, Rey A, Ruan H, Craine JM, Liang J, Zhou J, Luo Y (2016). Soil properties control decomposition of soil organic carbon: results from data-assimilation analysis. Geoderma.

[89] Yang R, Yang F, Yang F, Huang L, Liu F, Yang J, Zhao Y, Li D, Zhang G (2017). Pedogenic knowledge-aided modelling of soil inorganic carbon stocks in an alpine environment. Sci Total Environ.

[90] Yang Y, Fang J, Ji C, Ma W, Su S, Tang Z (2010). Soil inorganic carbon stock in the tibetan alpine grasslands. Global Biogeochem Cycle.

[91] Yang Y, Fang J, Smith P, Tang Y, Chen A, Ji C, Hu H, Rao S, Tan K, He J (2009). Changes in topsoil carbon stock in the Tibetan grasslands between the 1980s and 2004. Glob Change Biol.

[92] Ye X, Liu Z, Zhang S, Gao S, Liu G, Cui Q, Du J, Huang Z, Cornelissen JHC (2019). Experimental sand burial and precipitation enhancement alter plant and soil carbon allocation in a semi-arid steppe in north China. Sci Total Environ.

[93] Zamanian K, Zhou J, Kuzyakov Y (2021). Soil carbonates: the unaccounted, irrecoverable carbon source. Geoderma.

[94] Zamanian K, Kuzyakov Y (2019). Contribution of soil inorganic carbon to atmospheric CO_2_: more important than previously thought. Glob Change Biol.

[95] Zeng J, Guo T, Bao X, Wang Z, Sun J (2008). Effections of soil organic carbon and soil inorganic carbon under long-term fertilization. Soil Fertil Sci China..

[96] Zhang C, Liu G, Xue S, Sun C (2013). Soil organic carbon and total nitrogen storage as affected by land use in a small watershed of the loess plateau, China. Eur J Soil Biol.

[97] Zhang L, Zhao W, Zhang R, Cao H, Tan W (2018). Profile distribution of soil organic and inorganic carbon following revegetation on the loess plateau, China. Environ Sci Pollut Res.

[98] Zhang W, Wang X, Lu T, Shi H, Zhao Y (2020). Influences of soil properties and hydrological processes on soil carbon dynamics in the cropland of north China plain. Agr Ecosyst Environ.

[99] Zhang Y, Yu C, Xie J, Du S, Feng J, Guan D (2021). Comparison of fine root biomass and soil organic carbon stock between exotic and native mangrove. CATENA.

[100] Zhao W, Zhang R, Cao H, Tan W (2019). Factor contribution to soil organic and inorganic carbon accumulation in the loess plateau: structural equation modeling. Geoderma.

[101] Zhao W, Zhang R, Huang CQ, Wang BQ, Cao H, Koopal LK, Tan WF (2016). Effect of different vegetation cover on the vertical distribution of soil organic and inorganic carbon in the Zhifanggou watershed on the loess plateau. CATENA.

[102] Zhao X, Zhao C, Stahr K, Kuzyakov Y, Wei X (2020). The effect of microorganisms on soil carbonate recrystallization and abiotic CO_2_ uptake of soil. CATENA.

[103] Zhou G, Zhou X, Nie Y, Bai SH, Zhou L, Shao J, Cheng W, Wang J, Hu F, Fu Y (2018). Drought-induced changes in root biomass largely result from altered root morphological traits: evidence from a synthesis of global field trials. Plant Cell Environ.

[104] Zhu X, Si J, He X, Jia B, Zhou D, Wang C, Qin J, Liu Z (2024). Effects of long-term afforestation on soil water and carbon in the Alxa plateau. Front Plant Sci.

